# Three‐dimensional dosimetry of TomoTherapy by MRI‐based polymer gel technique

**DOI:** 10.1120/jacmp.v12i1.3273

**Published:** 2010-09-14

**Authors:** Yoichi Watanabe, N. Gopishankar

**Affiliations:** ^1^ Department of Therapeutic Radiology University of Minnesota Minneapolis MN USA; ^2^ All India Institute of Medical Sciences New Delhi India

**Keywords:** Helical TomoTherapy, IMRT, 3D dose measurement, polymer gel

## Abstract

Verification of the dose calculation model and the software used for treatment planning is an important step for accurate radiation delivery in radiation therapy. Using BANG3 polymer gel dosimeter with a 3 Tesla magnetic resonance imaging (MRI) scanner, we examined the accuracy of TomoTherapy treatment planning and radiation delivery. We evaluated one prostate treatment case and found the calculated three‐dimensional (3D) dose distributions agree with the measured 3D dose distributions with an exception in the regions where the dose was much smaller (25% or less) than the maximum dose (2.5 Gy). The analysis using the gamma‐index (3% dose difference and 3 mm distance‐to‐agreement) for a volume of 12 cm×11 cm×9 cm containing the planning target volume showed that the gamma values were smaller than unity for 53% of the voxels. Our measurement protocol and analysis tools can be easily applied to the evaluation of other newer complex radiation delivery techniques, such as intensity‐modulated arc therapy, with a reasonably low financial investment.

PACS numbers: 87.53Bn, 87.56Fc

## I. INTRODUCTION

A treatment for modern radiation therapy is designed by using a sophisticated computer program with advanced computation tools to compute absorbed dose in three‐dimensional (3D) space for given incident radiation beam intensities. Validation of dose distributions predicted by treatment planning software is an important step for high‐quality radiation therapy since the error in the calculated dose distributions would lead to incorrect dosages to both the malignant tumor cells (or the target) and surrounding healthy normal organs. Using 3D dose measurement tools – in particular, polymer gel dosimeter – we can obtain complete 3D dose distributions.^(^ ^1^ ^,^ ^2^ ^)^ In spite of the promise for such an enhanced dosimetric capability for last 15 years, clinical applications of the polymer gel dosimetry is lacking for four reasons: the inherent complexity of the procedures, the cost of both the polymer gel and the scanning tool, absence of analysis software readily available, and a lack of clear and urgent necessity to have true 3D visualization of delivered dose distributions.

In this article, we presented the results of 3D dose measurements using BANG3 polymer gel in combination with a 3 Tesla (3T) magnetic resonance imaging (MRI) scanner for radiation therapy of a prostate cancer patient by a helical TomoTherapy Hi·ART II treatment system (TomoTherapy Inc., Madison, WI).^(^ ^3^ ^)^ As a part of patient‐specific quality assurance processes for TomoTherapy, a point dose and dose distribution on a plane (usually on a coronal plane) are measured using an ionization chamber and a EDR2 film (Eastman Kodak Company, Rochester, NY) inserted in a special cylindrical solid phantom (called “cheese” phantom). Beyond those measurements routinely performed at all TomoTherapy user sites, there are only a few publications available for experimental verification of the dose distributions computed by using the TomoTherapy treatment planning software. There have been comprehensive verification in two‐dimension (on a plane or a cylindrical surface) using radiographic films.^(^ ^4^ ^,^ ^5^ ^)^ In our study, we performed a true 3D verification of computer‐predicted dose distributions using a polymer gel dosimeter. We have developed all the tools necessary to accomplish the measurements and analyses in our radiation oncology clinic using commercially available polymer gel phantoms, and our procedure can be easily adapted to 3D dose measurements for complicated radiation therapy in other radiation oncology departments.

## II. MATERIALS AND METHODS

### A. TomoTherapy treatment case

At University of Minnesota we use a helical TomoTherapy system Hi·ART II for IMRT treatments. TomoTherapy delivers intensity‐modulated 6 MV photon beams with three different field widths in the cranial‐caudal direction (1.05 cm, 2.5 cm and 5 cm). A treatment length is covered by moving the treatment couch while the gantry head is rotating. For this study, we chose one of our prostate cancer treatment cases. Note that the polymer gel dosimetry procedure we used for this study can be applied to any treatment site in which the treatment volume is not larger than the size of the phantom. A total dose of 30.6 Gy was delivered with a daily fraction of 1.8 Gy to the planning target volume (PTV), of which size was approximately 6 cm wide in the left‐right direction (X direction), 5 cm high in the anterior‐posterior direction (Y direction), and 6 cm long in the cranial‐caudal direction (Z direction) with the total volume of $137.7\;{\text{cm}}^{3}$. The treatment plan was optimized to reduce the doses to rectum, bladder and femoral heads. Some of the physical parameters for the dose delivery design were as follows: the pitch =0.30, the modulation factor =2.6 and the field width =2.5 cm. A treatment‐specific plan for quality assurance called as dynamic QA (DQA) was created with the cheese phantom using the approved beam intensity pattern. For the DQA plan, a point dose and a planar dose distribution on a coronal plane were measured using a cylindrical ionization chamber and an EDR2 film. The measured dosimetric data were compared with those computed by the treatment planning software. The treatment plan used for this study passed the DQA test (a point dose error smaller than 3% and the computed planar dose distribution agreeing with the film measured distribution) and was used for the actual treatment. Note that, at our clinic, the gamma index analysis is not performed for a patient‐specific DQA.

### B. Polymer gel dosimeter

There are many variations of polymer gels developed by investigators all over the world since the first practical polymer gel was invented and developed in early 1990s.^(^ ^2^ ^)^ In fact, generating one's own polymer gel is not very difficult and requires the minimum facility with relatively inexpensive equipments. For a practical reason, however, we purchased BANG3‐Pro‐2,^(^ ^6^ ^)^ a modified version of BANG3 polymer gel from MGS Research (Madison, CT), which is currently the only commercial vendor who markets the polymer gel for radiation dosimetry. The polymer gel was contained in an 18 cm diameter and 18 cm long cylinder made of 3 mm thin acrylic walls. Herein we call this a BANG3 cylindrical phantom. The whole container was covered with a black paper to prevent sunlight from damaging the polymer gel. For dose response calibration, we used 10 small tubes (1 cm diameter and 8 cm long glass tubes) filled with the BANG3 polymer gel, which was made at the same time as the gel used to fill the larger cylindrical phantom. The temperature environment where the polymer gel is stored for a longer period is an important factor affecting the response characteristics to radiation. Hence, we paid special attention on this issue by placing the BANG3 containers in a refrigerator before use and in specific rooms to equilibrate the gel temperature with the room temperature during the irradiation and the MR scanning.

The calibration tubes were placed in a scanning water tank (37 cm×37 cm×37 cm) and irradiated with opposed parallel 6 MV photon beams from an Elekta Synergy accelerator (Elekta, Stockholm, Sweden). Using a 20 cm by 20 cm open field, a relatively uniform dose distribution was achieved throughout the entire calibration tubes. For the response calibration, we gave 0.5, 1.0, 2.0 (2 tubes), 3.0 (2 tubes), 4.0, and 5.0 Gy to the tubes. A pair of nonirradiated calibration tubes was used as a control. The BANG3 cylindrical phantom was irradiated with TomoTherapy using the DQA plan for the prostate treatment case described in the previous section.

One day after the irradiation, the BANG3 cylindrical phantom and the calibration tubes were scanned with a 3T MRI scanner, MAGNETOM Trio (A Tim System) (Siemens Medical Solutions, Erlangen, Germany) at the Center for Magnetic Resonance Research (CMRR). The phantom was placed in a 12‐channel head matrix coil by aligning the MRI‐visible fiducial markers on the phantom with the in‐room lasers for the scan. First, to determine the repetition time (TR), the spin‐lattice relaxation time (T1) of the BANG3 polymer gel was measured using a gradient echo technique. The measured T1 value of the BANG3 gel was 860 ms. Then we measured the spin‐spin relaxation rate (R2) distribution by applying the multi‐spin echo pulse sequence available on the Siemens MRI scanners (designated as “cp_mc” on the machine), which is a variation of the standard Car‐Purcell‐Meiboom‐Gill (CPMG) pulse sequence. The imaging parameters were specifically selected for the polymer gel as follows: the field‐of‐view (FOV)=256 mm ×256×256 mm, 256  ×256 pixels, 2 mm slice thickness without gap, TR=7000 ms (approximately 8×T1), echo spacing ΔTE=13.6 ms, and the number of echoes = 32. Forty‐five slices were acquired during one scan, which took about 90 minutes to complete. An interleaved slice acquisition method was chosen to minimize the interference between nearby imaging slices.

To calculate the dose deposited in the BANG3 cylindrical phantom by the TomoTherapy DQA plan delivery, the phantom was scanned with a CT scanner, Philips Brilliance Big Bore CT (Philips Medical, Eindhoven, The Netherlands) after the MRI scan. The CT data were imported into the TomoTherapy treatment planning computer and a DQA plan for the TomoTherapy prostate treatment was recreated for the BANG3 cylindrical phantom.

### C. Dose reconstruction and analyses

The MRI data of the BANG3 cylindrical phantom as well as the calibration tubes were processed using an in‐house MATLAB (The MathWorks, Inc., Natick, MA) based program, which was named as ATOM. This program calculated the R2 values of all pixels from 32 images taken at 32 different echo times for all 45 slices (i.e., total 1440 slices.) It was assumed that the echo signal decays exponentially in time. The maximum likelihood estimation method was used to estimate the decay constant (i.e., R2) in the exponential decay equation. To improve the accuracy of the R2 estimation, a new algorithm that automatically selects the number of echo signals useful for the estimation was introduced.^(^ ^7^ ^)^ Note that the first echo signal was omitted from the R2 estimation.

The R2 values obtained above for the calibration tubes were used to determine the correspondence between the dose (D) absorbed in the BANG3 polymer gel and the R2 value. The calibration data were fitted with a linear equation as represented as:

(1) D=a R2+b

This equation then was applied to the R2 data of the BANG3 cylindrical phantom to convert the R2 values to the dose values. The final measured dose data were stored as a 256×256×45 3D matrix. Note that the voxel size for the measured dose distribution was 1 mm×1 mm×2 mm.

The dose distribution data calculated by the TomoTherapy treatment planning software for the BANG3 cylindrical phantom were imported into the CERR program (Advanced Radiotherapy Treatment Planning Group, Washington University, St. Louis, MO), by which we converted the dose data written in a TomoTherapy‐specific format into a standard matrix format readable by a MATLAB program. The calculated dose matrix size was 256×256×66 and the voxel size (or 3D grid size) was 1 mm×1 mm×3 mm. Note that the 3 mm grid size of the calculated dose in the Z direction was determined by the slice thickness used for the CT scan of the BANG3 cylindrical phantom.

3D dose comparison of the measured and calculated doses was accomplished by using in‐house MATLAB based programs PG2DCMP and PG3DCMP, which first recomputed the measured dose values at the 3D spatial grid points of the computed dose matrix (i.e., 256×256×66=4,325,376 voxels) by using a 3D linear interpolation algorithm. The program was then used to plot the slice‐by‐slice isodose distributions, and calculate the percentage dose differences and the gamma‐index values.

The gamma index γ at a spatial point *r* is defined by^(^ ^8^ ^)^

(2) $$\gamma (r)={\text{min}}_{i}\Gamma (D_{m}(r_{1}),\;D_{c}(r))$$

where

(3) $$\Gamma (D_{m}(r_{1}),D_{c}(r))=\sqrt{{\left(\frac{D_{m}(r_{1})-D_{c}(r)}{D_{\text{ref}}8D}\right)}^{2}}+{\left(\frac{r_{1}-r}{\Delta }\right)}^{2}$$

and $D_{m}(r)$ and $D_{c}(r)$ are the measured dose and calculated dose at point *r*. The minimum is taken by exhaustively searching for the measured dose at points $r_{i}$ near *r*. A new algorithm was developed to compute the gamma index in 3D within a reasonable computer time. The algorithm searches for the minimum of the function Γ in a 2 cm×2 cm×2 cm cube consisting of 20×20×20 grid points, whose center is at the point *r*, at which the γ index is being calculated. As the tolerances for the gamma index calculation we chose 3% dose difference (δD) and 3 mm distance‐to‐agreement (Δ). Hence, the choice of the 2 cm side length for the gamma index search is reasonable. $D_{\text{ref}}$ was set to 2 Gy, which is larger than the prescription dose of 1.8 Gy because the BANG3 cylindrical phantom was smaller than the actual size of the patient, leading to larger doses in the phantom than the doses delivered to the patient. It is noted that the proposed algorithm is similar to a recently published method.^(^ ^9^ ^)^

The uncertainty of measured dose values was estimated by propagating the uncertainties of parameters in the calibration equation, Eq. (1), and the R2 values. We can estimate the uncertainties of the coefficients, the slope *a* and the offset *b*, in Eq. (1) from the calibration data. With the estimated uncertainty of R2, we can calculate the uncertainty of the dose from the following equation:^(^ ^10^ ^)^

(4) $$\frac{\Delta D}{D}=\sqrt{{\left(\frac{aR2}{D}\frac{\Delta a}{a}\right)}^{2}+{\left(\frac{b}{D}\frac{\Delta b}{b}\right)}^{2}+{\left(\frac{aR2}{D}\frac{\Delta R2}{R2}\right)}^{2}}$$

Here we express the uncertainty of parameter α by Δ α /α.

The uncertainty of the R2 values can be estimated by measuring the R2 values in a number of pixels and calculating the standard deviation of the measured R2 values. To estimate the uncertainty of the R2 values at all voxels, we used a theoretical formula called the Cramer‐Rao lower bound (CRLB) of the variance of an estimator.^(^ ^11^ ^)^

## III. RESULTS

### A. Dose response calibration

**Figure 1:**
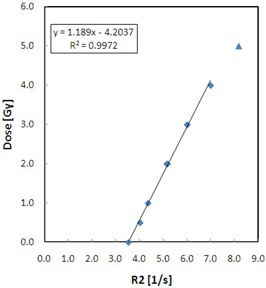
Dose response calibration data represented as the absorbed dose vs. R2 value. A linear regression curve is displayed. The standard deviation of the R2 value was as small as the size of the symbol, or approximately 1%.

Figure 1 shows the dose calibration data. To derive a linear regression equation we omitted the data point corresponding to the 5 Gy dose. This is acceptable because the maximum dose to the phantom used for the treatment plan evaluation did not exceed 2.5 Gy. The regression analysis lead to the following values: the slope a=1.19(±3.21%) and the offset b=−4.20(±2.15%). The coefficient of the determinant R2 was 0.9972, which indicated an excellent linear fit for the dose range of interest.

### B. 3D dose comparison

We obtained complete 3D dose distribution data for both measurements and calculations. It is noted that the measured dose values were scaled by multiplying the original dose with a factor of 0.77. This dose scaling factor was determined by comparing the dose measured by the BANG polymer gel using the aforementioned calibration data with the dose calculated by the TomoTherapy treatment planning software in a relatively uniform dose region inside the target volume. An independent point‐dose measurement using a small volume ionization chamber with the cheese phantom already had shown that in this region the calculated dose agreed with the measured dose within 1%. Such a dose scaling procedure is acceptable for relative dosimetry,^(^ ^10^ ^)^ and it is needed to adjust the difference in radiation response of the gel contained in different sizes of containers in the current study, for which we used small tubes for calibration and a large phantom for dose comparison measurements. The response depends on the size of container because of the difference in temperature rise during an MRI scan and potentially higher oxygen contamination in smaller tubes.^(^ ^12^ ^)^

**Figure 2:**
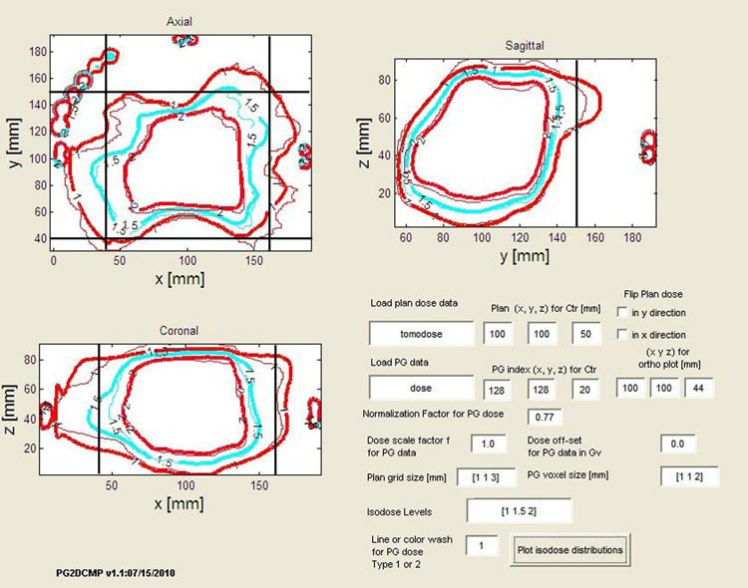
The overlays of measured (thick) and computed (thin) dose distributions on three orthogonal planes for isodose lines of 1, 1.5 and 2 Gy. The axial, sagittal and coronal planes are at Z=44 mm, X=100 mm, and Y=100 mm, respectively, The Figure shows the GUI window of the PG2DCMP program. Solid straight lines on the three planes indicate the boundaries of the volume V used for the dose comparison, such as gamma index analysis.

**Figure 3:**
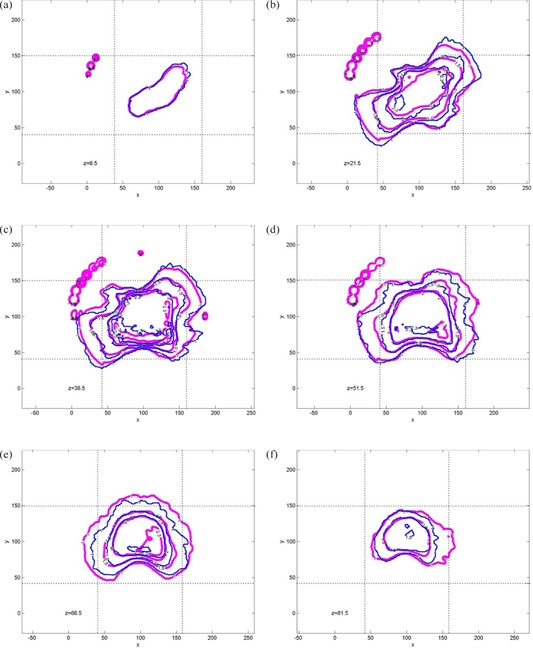
Comparison of measured and computed dose distributions on transverse planes: (a) z=6.5 mm, (b) z=21.5 mm, (c) z=36.5 mm, (d) z=51.5 mm, (e) z=66.5 mm, and (f) z = 82.5 mm. Isodose lines for 1, 1.5, 2, and 2.3 Gy of measurements (thick magenta) and calculations (thin blue) are plotted. The units of x‐ and y‐axes are in mm. The dotted straight lines indicate the area where gamma indices were calculated and displayed in Fig. 4.

Presenting the enormous data available from a 3D study in a meaningful way is a challenging task. Here we limit our presentation to a few figures. Isodose distribution comparison is presented in Fig. 2 and Figs. 3(a) to 3(f). Figure 2 shows the 2D dose distributions on three orthogonal planes. The graphics in Fig. 3 overlay isodose contours of the measured and calculated data on six slices in the Z direction. The isodose plots shown in Fig. 2 indicate a fair agreement of the 1, 1.5, and 2 Gy isodose curves between measured and calculated doses. It is noted, however, that the length of high‐dose volume indicated by the 1 Gy isodose curve by calculations is slightly wider in the Z direction than that by measurements as seen on both coronal and sagittal planes. For example, look into the 1 Gy isodose curves near the top edge of the plots above the Z=80 mm region on the isodose plot on the sagittal plane in Fig. 2. Figure 3 compares the isodose distributions on six transverse planes along the Z direction. These Figures also show a good agreement of 1, 1.5, 2, and 2.3 Gy isodose curves between the measured and calculated doses; but one can notice larger differences of 1 Gy isodose curves compared with higher isodose curves.

The remaining results presented in this article were obtained by limiting the volume of dose comparison to a volume large enough to include the high‐dose region, but excluding the outside of the BANG3 cylindrical phantom (i.e., the volume V: a 12 cm×11 cm×9 cm volume surrounding the target volume). For clarity, in Figs. 2 and 3, straight lines were drawn to indicate the volume V used for the analysis.

**Figure 4:**
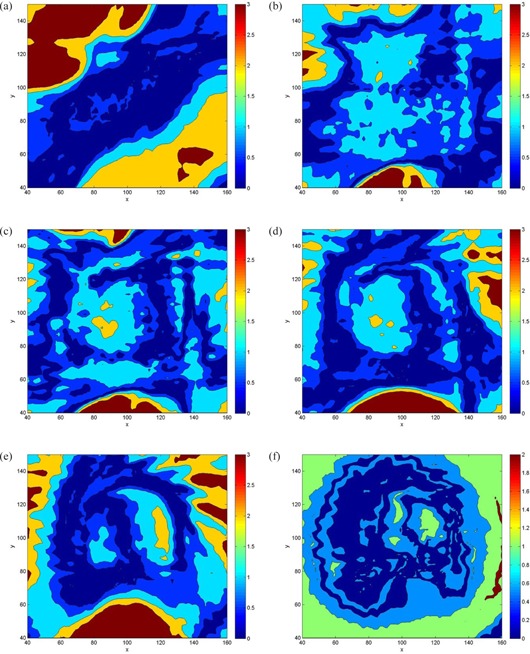
Gamma index distributions plotted on transverse planes: (a) z=6.5 mm, (b) z=21.5 mm, (c) z=36.5 mm, (d) z=51.5 mm, (e) z=66.5 mm, and (f) z = 81.5 mm. The criteria for the gamma index calculations were 3% dose difference and 3 mm distance‐to‐agreement. The units of x‐ and y‐axes are in mm.

**Figure 5:**
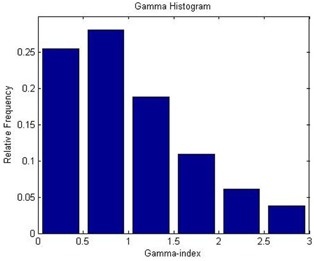
Histogram showing the gamma index distribution. The criteria for the gamma index calculations were 3% dose difference and 3 mm distance‐to‐agreement.

Figures 4(a) to 4(f) are gamma index plots for the corresponding slices shown in Fig. 3. The gamma index is smaller than 1 for the majority of the high‐dose regions. Larger gamma index values are observed outside the target volume. The gamma index analysis can be summarized by using a histogram shown in Fig. 5, which indicates the portion of the voxels meeting the tolerance criteria (or γ≤1). About 53% of voxels included in the gamma index calculations had γ smaller than unity.

**Figure 6:**
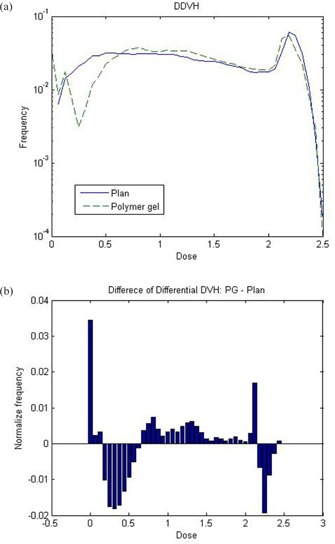
Differential dose‐volume histogram (DDVH) (a), and difference in differential dose‐volume histograms (b) for measured and computed dose distributions (DDDVH).

**Figure 7:**
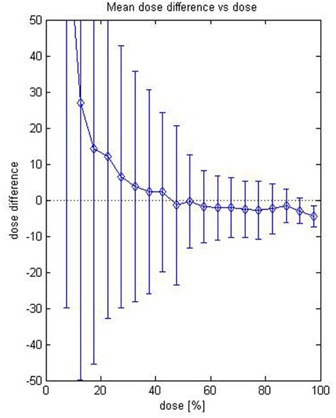
Mean dose difference vs. dose level. The dose in the horizontal axis was normalized to 100% at the maximum dose of 2.5 Gy.

Since we have a complete 3D dose data, we can do statistical analysis including all dose points in the 3D space. Some of such analyses are now presented for the volume V. Figure 6(a) shows the differential dose‐volume histogram (DDVH) indicating the (relative) number of voxels with a specific dose for the measured and calculated dose data. The DDVH curves are different from each other and the difference depends on the dose range. Note a significantly large difference in the dose range between 0.2 Gy and 0.5 Gy. This is clearly seen in Fig. 6(b), which was obtained by subtracting the number of voxels in dose bins for the measured dose from those for the calculated dose. This histogram, the difference in DDVH, is denoted as DDDVH. This figure clearly shows that the measured dose was smaller than the calculation in the dose range between 0.2 Gy and 0.6 Gy and for the dose above 2.1 Gy; meanwhile, the measured dose was greater for the doses between 0.6 Gy and 2.1 Gy as well as below 0.2 Gy. Figure 7 shows the mean dose difference as a function of dose with the standard deviation. Note that 100% in the horizontal axis corresponds to the maximum dose in the calculated doses (or 2.5 Gy). The Figure indicates that the mean dose difference between the measurement and the calculation is smaller than 5% for the dose larger than 50% (or 1.25 Gy) with a standard deviation of less than ±10%. But, both the difference and the standard deviation increase sharply as the dose decreases toward zero. These results indicate that the calculated doses were significantly different from the measured doses for lower doses, notably for less than 25% (or 0.6 Gy) of the maximum dose.

### C. Uncertainty analysis

**Figure 8:**
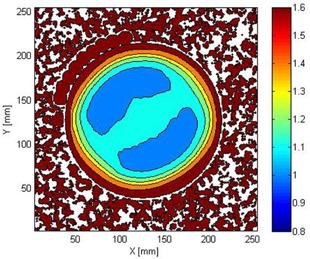
The spatial distribution of the uncertainty of the measure R2 values plotted on a transverse plane (z=51.5 mm), which corresponds to the plane in Figs. 3(d) and 4(d). The color bar on the right indicates the colors corresponding to percentage uncertainty values.

The presentation of measurement results and its comparison to calculation is not complete without discussing the measurement uncertainty. According to the standard procedure for uncertain estimation,^(^ ^13^ ^)^ there are two types of uncertainties, type A and type B. The type A uncertainty is associated with the statistical error, which can be obtained from measured data. For the current study, only MR signal intensity data can provide the type A uncertainty. From the analysis of the MR image data, we estimated one standard deviation of the measured signal was 1% for the shortest echo time (or at 13.6 ms), or the signal‐to‐noise ratio (SNR) was 60. Note that uncertainty increases as the signal decays in time. By applying the CRLB formula, we estimated the uncertainty of the R2 value. The result suggests that the uncertainty of the estimated R2 is about 1.0% in the high‐dose region. Figure 8 shows the R2 uncertainty distribution on a transverse plane of Z=51.4 mm, which corresponds to the plane in Figs. 3(d) and 4(d).

By simply using the uncertainty values of 3.21%, 2.15%, and 1.0% for *a*, *b*, and R2, respectively, in Eq. (4), we obtained the uncertainty for the measured dose, which non‐linearly decreases with increasing dose (i.e., 30% at 0.5 Gy and 5.8% at 4 Gy). It is noted, however, that the uncertainty of doses can be estimated by ignoring the uncertainties of the coefficients *a* and *b* in the calibration equation Eq. (1) when only relative dose distribution is considered. Under this assumption, the uncertainty of the relative dose is $\sqrt{2}$ times the uncertainty of R2.^(^ ^10^ ^)^ Hence, the uncertainty of measured dose distribution is about 1.5% with one standard deviation (or 3.0% for two standard deviations or the 95% confidence level) for this study.

## IV. DISCUSSION

### A. Physical parameters for comparison

3D dosimetric comparison can be done using the techniques used for comparison of treatment plans, dose computation algorithms and different radiation delivery techniques. Comparing isodose lines on many planes are one of the techniques. Another common physical quantity for those purposes has been dose‐volume histograms for both the target and critical structures. For the current study, we presented the differential dose‐volume histogram (DDVH) and the difference in the differential dose‐volume histogram (DDDVH) as shown in Fig. 6. DDDVH is particularly useful to examine the difference more clearly than just plotting two DVH or DDVH curves. We used a parallelepiped volume for the histogram calculations. We could not compare the DVH specific to the structures because the current version of our in‐house programs cannot use the spatial location data of the structures for the DVH analysis.

The gamma index is widely accepted as the physical parameter for dosimetric comparison – in particular, comparing measured dose and computed dose. One drawback of this approach is a lack of a clear consensus for an acceptable tolerance criterion of the dose difference and the distance‐to‐agreement. Furthermore, investigators have used various passing criteria for the percentage of gamma index values smaller than unity.^(^ ^4^ ^)^

The gamma index analysis of the current 3D polymer gel dosimetry study showed a passing rate of 53%, which is lower than the passing rate considered to be acceptable for clinical deliveries (i.e., usually above 90%). As discussed in the Methods and Material section, the treatment plan used for this study passed our routine patient‐specific pretreatment QA (or DQA). However, it should be pointed out that there are two differences between the current 3D analysis and the 2D analysis done for DQA. For the DQA, we place a film in the cheese phantom and measure the 2D dose distribution on single coronal plane approximately placed at the center of the high dose region. Only line dose profiles of measurements and calculations are visually compared to make judgment. Secondly, we do not do the gamma analysis of the dose distribution normally in our clinic. We did retrospective analyses of the 2D dose distribution taken by the film placed on a coronal plane. Gamma index histogram was computed and the results showed that about 80% of pixels on the plane passed the gamma criteria (3% dose difference and 3 mm distance‐to‐agreement). Considering that this is only for one plane, the passing rate of 53% for the 3D analysis using the BANG3 polymer gel is not a surprise. Another factor affecting the gamma index value is the selection of $D_{\text{ref}}$ in Eq. (3), for which we chose 2 Gy. When the maximum dose of 2.5 Gy was chosen for the analysis, in fact the gamma index values became smaller, resulting in a 59% passing rate. The current results strongly suggest a need for a true 3D dosimetry.

### B. Practical consideration

For this study, we purchased BANG3‐PRO‐2. At our institution, the user fee for a two‐hour MRI scan session was $500. It is expected that more and more radiation oncology departments will acquire an MRI scanner for radiation therapy treatment planning and treatment monitoring in the near future; hence, MRI will become more accessible without fee for medical physicists, in particular, for clinical physics QA applications. For this study, data analysis software was developed in‐house on the MATLAB platform. As the polymer gel technique becomes more widely used, a commercial vendor might offer software capable of doing the analysis presented in this article. Certainly such software can be used not only for polymer gel dosimetry but also for other film‐based dosimetry. Hence, there will be no large extra expenditure involved in the analytical tool. Consequently, the only necessary expense will be most likely the cost of the polymer gel, which is currently $75 per L (or $340 for 4.5 L plus $100 for a reusable container), and certainly the price is expected to fall when the demand for the polymer gel increases. This price is comparable to purchasing 25 EDR2 films (currently $315). Notice that the polymer gel is much easier to use for 3D dosimetry applications than using many films placed in a solid phantom. Therefore, more and more polymer gel dosimeter applications are expected for commissioning and validating new treatment planning software and new sophisticated radiation delivery technologies (notably, the intensity‐modulated arc therapy) in the coming years.

The time needed to complete a 3D measurement using a polymer gel dosimeter is at least three days. Once one receives a new phantom, it is desirable to have about one day before irradiation of the phantom, then another day between the irradiation and an MR scan. The analysis can be done quickly by an experienced physicist but still requires one day of work. Hence, the measurement time is relatively short when one recognizes that a complete 3D dose distribution can be obtained after this three‐day procedure.

## V. CONCLUSIONS

The main goal of this article was to present how a clinically useful polymer gel dosimetry is accomplished using tools available in radiation oncology clinics. We used a commercially‐available polymer gel BANG3 for 3D dose verification of one of TomoTherapy treatment plans. The phantom was scanned with a 3T MRI scanner using a built‐in CPMG pulse sequence with image acquisition parameters optimized for estimation of R2 values. In‐house computer programs developed on the MATLAB platform were used for the analyses. The whole measurements and analyses sequence can be completed within three days. The measurement gave us a full 3D dose distribution for the volume of 256 mm×256 mm×90 mm (the spatial resolution of 1 mm×1 mm×2 mm) with a small uncertainty (<2%) of the estimated dose values. Comparison of the measured dose distributions with the computed dose distributions showed a good overall agreement; at the same time, we have discovered there is a large discrepancy existing at certain regions in the 3D space. In particular, for doses lower than 25% of the maximum dose (or 2.5 Gy for this study), the agreement degraded. This issue warrants further investigation. We have now a strong confidence that the measurement procedure used for this study can be applied to commissioning new treatment planning software and new treatment delivery technologies (especially those using the intensity‐modulated arc delivery techniques whose delivered dose is below 3 Gy), and will provide clinically useful measurement precision for reasonable material and equipment cost.

## ACKNOWLEDGEMENTS

The first author would like to thank Professor Hitoshi Kubo at University of Tokushima, Japan for helping him to set up the MRI protocol used for this study. CMRR was funded by NIH grants (P30 NS057091, P41 RR008079). Continuing support to the second author by Dr. S. Vivekanandhan of AIIMS is greatly appreciated.
